# Objective Physical Activity by Frailty Status: A Cross-Study Comparison

**DOI:** 10.1093/geroni/igaf122.642

**Published:** 2025-12-31

**Authors:** Jennifer Schrack, Lacey Etzkorn, Sunan Gao, Brian Buta, Anis Davoudi, Qian-Li Xue, Karen Bandeen-Roche, Amal Wanigatunga

**Affiliations:** Johns Hopkins Bloomberg School of Public Health, Baltimore, Maryland, United States; Johns Hopkins Bloomberg School of Public Health, Baltimore, Maryland, United States; Johns Hopkins University, Baltimore, Maryland, United States; Johns Hopkins University, Baltimore, Maryland, United States; Johns Hopkins University, Baltimore, Maryland, United States; Johns Hopkins University, Baltimore, Maryland, United States; Johns Hopkins Center on Aging and Health, Baltimore, Maryland, United States; Johns Hopkins Bloomberg School of Public Health, Baltimore, Maryland, United States

## Abstract

Physical activity (PA) is paramount to delaying the onset and progression of frailty. Current understanding of how PA differs by frailty status is mostly limited to select populations and/or summary measures of time spent at various intensities and fails to account for the typical amount and patterns of daily PA in which most older adults engage. We investigated differences in daily PA among 3,558 older adults aged ≥70 years (mean=79.2±5.7years, 52.9% female) from five cohorts: ARIC, BLSA, NHATS, STURDY, and ACHIEVE. PA was assessed using identical 24-hour/7-day wrist-worn accelerometry protocols and defined using measures of activity volume (min/day), fragmentation (% probability of transitioning from an active-to-sedentary state), and endurance (minutes/active bout). Frailty was defined as having ≥3 of low self-reported walking for exercise or sport activities, low grip strength, slow walking speed, exhaustion, and unintentional weight loss; having 1-2 was considered prefrail. Across cohorts, 395(11.1%) were frail, 1,975(55.5%) prefrail, and 1,188(33.4%) robust. Robust older adults averaged 406±107 active min/day, 24.2±5.8% fragmentation, and 4.4±1.1 minutes/active bout. Frail and prefrail older adults had lower activity volume [317±131 and 377±115 active minutes/day, respectively], were more fragmented [31.6±11% and 26.3±7.1%, respectively], and had lower average bout length [3.6±1.3 and 4.1±1.1 min/bout, respectively], comparatively. Time-of-day analyses indicated, relative to the robust group, frail and prefrail older adults were more active overnight (12-5am) and less active during waking hours (8am-8pm). These results represent a first step in establishing normative values for objective PA across the frailty spectrum, providing a path towards earlier detection and treatment.

